# Evaluation of physical properties of rice used in traditional Kyrgyz Cuisine

**DOI:** 10.1002/fsn3.746

**Published:** 2018-08-13

**Authors:** Martina Nádvorníková, Jan Banout, David Herák, Vladimír Verner

**Affiliations:** ^1^ Faculty of Tropical AgriSciences Czech University of Life Sciences Prague Praha – Suchdol Czech Republic; ^2^ Faculty of Engineering Czech University of Life Sciences Prague Praha – Suchdol Czech Republic

**Keywords:** cooking time, imported varieties, Kyrgyz rice, physical properties, traditional cuisine

## Abstract

Eight staple rice cultivars consumed in Kyrgyzstan were evaluated for physical properties in this study. The dimensions of investigated grains correspond to 5.29–6.99 mm for length, 2.52–3.10 mm for width, and 1.88–2.13 for thickness. Equivalent diameter was in range of 3.14 – 3.47 mm, surface area took 25.35–31.90 mm². The sphericity analysis values varied from 0.480 to 0.559, aspect ratio from 0.39 to 0.55, volume of the grain was measured in range from 16.25 to 22.02 mm^3^, bulk density values were 0.77–0.87 g/cm^3^, and solid density from 1.17 to 1.41 g/cm^3^. The porosity of grain was equal to 28.27–39.83%, thousand kernel weight correspond to 19.67 to 27.15 g, rupture force of grain was measured in range of 63.47–155.50 N, color characteristic varied in parameters L*, a* and b*, 37.58–72.19, –0.22–10.17, and 9.65–21.12, respectively. Optimum cooking time ranged from 19.33 to 33.00 min. The water uptake ratios for 30 min of soaking were 1.21–1.28, 1.18–1.45, and 1.14–1.57 for 30, 45, and 60°C, respectively. While the water uptake ratios for 60 min of soaking were 1.22–1.42, 1.19–1.54, and 1.25–1.75 for 30, 45, and 60°C, respectively. Optimal cooking time showed that imported varieties needed lower interval for full grain cooking compared to the local Kyrgyz varieties. It was found that Kyrgyz rice varieties staying more firm after cooking as compared to imported varieties and therefore more suitable for the local traditional dish such as plov.

## 1 INTRODUCTION

Distinction of rice grain quality indicates its market value and has also a crucial role in popularity in local cuisine or increase potential adoption of new varieties. Main quality traits encompass physical appearance, cooking and sensory properties, textural characteristics, and nutritional value. The valuing of each trait or attribute by consumers varies according to local cuisine and culture (Fitzgerald, McCouch, & Hall, 2009). Grain quality indicators such as rupture force and color have great importance in the food industry. Differentiation of the varieties helps to improve the consumer's acceptability and popularity of the particular rice cultivar, and the knowledge of physical properties of grain materials is also useful for designing appropriate machineries for grain processing operations such as sorting, drying, heating, cooling, milling, and optimization connected to each specific variety (Mir, Bosco, & Sunooj, 2013).

Rice (*Oryza sativa* L.) represents one of the most important and highly nutritious food crops contributing to one‐quarter of global per capita dietary energy intake. There are plenty of studies documenting the physical and qualitative attributes of rice grain for different cultivars particularly from South‐East Asia, main rice cropping area (Hori, 2016; Kaur, Panesar, & Bera, 2011; Mir et al., 2013; Tran et al., 2012). Nevertheless, considerably less attention is given to rice grain quality research in other parts of the world, such as Central Asia, where rice has historically being recognized as the most popular staple food after wheat with important cultural role in local cuisine (Kochkunov, 2010; Zanca, 2003). Local rice varieties are still cultivated in this region. However, there is lack of scientific information about their qualitative and sensory attributes.

Typical country from Central Asia where rice plays important role in both food security as well as in local traditional cuisine is Kyrgyzstan. Based on Food and Agriculture Organization (FAO) statistics, the total cultivated area of rice is around 8,000 ha and annual production equal to 28,230 tonnes and per capita consumption equal to 7.3 kg (Food and Agriculture Organization (FAO), 2014; Food and Agriculture Organization (FAO), 2016). As mentioned earlier, rice plays a crucial role in the cuisine of central Asian countries including Kyrgyzstan and represents a main ingredient in traditional festive dish “plov” preparation (Kochkunov, 2010; Kokaisl, 2013; Vlkova et al., 2015; Zanca, 2003). The most popular Kyrgyz local variety of rice is called Ozgon rice, grown in the Osh province and being famous for its extraordinary taste. The grains differ from other rice varieties by red‐brown color and longitudinal brown stripes on the grain body due to the unique climate, mineral composition of the soil as well farming conditions (Smanalieva, Salieva, Borkoev, Windhab, & Fischer, 2015). As there is according to our best knowledge, lack of scientific studies describing qualitative properties and attributes of local rice varieties in Central Asian countries particularly Kyrgyzstan, the main objective of the study was to examine and evaluate the physical properties of eight staple rice varieties consumed in that region. This study also determines the differences in physical, textural, and cooking properties between studied cultivars and implements them into the rice system scheme.

## 2 MATERIALS AND METHODS

### 2.1 Plant material

The dried rice kernels were purchased in February 2015 from the city market in Osh, South Kyrgyzstan. In total, eight different rice cultivars were bought, of which were five cultivars local varieties (Ozgon Champion, Batken, Ozgon Kakyr, Ozgon Uchuk and Ozgon Cerza), and three were imported varieties (Elita Krasnodar Russia from Russia, Pakistan from Pakistan, and Kapchygai Kazakhstan from Kazakhstan). The samples were collected from three different vendors, to sustain average sample quality of each variety (in average amount of 700 g). Cultivar samples were cleaned from small particles (as stones and dried weeds) and kept in closed plastic bags in room temperature till the laboratory tests. Only good quality rice grains were used during the analysis, and each measurement was taken in several/adequate number of repetitions to ensure significant result. The physical analysis was carried out in the Laboratory of Food Technology of Czech University of Life Sciences Prague, Faculty of Tropical AgriSciences between March, 2015 and February, 2016.

### 2.2 Length, width and thickness

The length L (mm), width W (mm), and thickness T (mm) of the rice kernels were determined by use of Vernier caliper with accuracy of 0.01 mm. Rice kernels were randomly selected, and each of their dimension was measured and recorded 20 times on each cultivar.

### 2.3 Equivalent diameter

Equivalent diameter *D*_*e*_ (mm) was determined using a following equation (Mohsenin, 1986):

$$D_{e}\;=\;{\left(L\frac{{\left(W\;+\;T\right)}^{2}}{4}\right)}^{\frac{1}{3}}$$

### 2.4 Surface area

For calculation of surface area *S* (mm^2^) were used previously recorded variables length, width, and thickness. The formula for surface area was described by Mohsenin (1986) and Jain and Bal (1997) as the following:

$$S\;=\;\frac{\pi \;\times \;B\;\times \;L^{2}}{2\;\times \;L-B}$$

where *B* = (*W* × *T*)^1/2^ (mm) is a function of width and thickness.

### 2.5 Sphericity

The sphericity of rice *φ* (−), was defined by the formula mentioned by Mohsenin (1986):

$$\varphi \;=\;\frac{{\left(L\;\times \;W\;\times \;T\right)}^{1/3}}{L}$$

### 2.6 Aspect ratio

The aspect ratio *R*_*a*_ (−) was calculated using formula (Varnamkhasti et al., 2008):

$$R_{a}\;=\;\frac{W}{L}$$

### 2.7 Volume

Volume of grain *V* (mm^3^) was calculated by the formula mentioned by Jain and Bal (1997) as following:

$$V\;=\;\frac{1}{4}\;\times \;\left[\left(\pi /6\right)\;\times \;L\;\times \;{\left(W\;\times \;T\right)}^{2}\right]$$

### 2.8 Bulk density

The bulk density *ρ*_*b*_ (kg/m^3^) was calculated according to equation mentioned by Fraser, Verma, and Muir (1978) when the 200 ml beaker was filled with the rice up to 100 ml sign and then the mass of rice grains was weighed. The weight of the rice was divided with the volume of the beaker (100 ml). The procedure was repeated five times:

$$\rho_{b}\;=\;\frac{M_{g}}{V_{b}}$$

where *M*_g_ (g) is mass of the grain and *V*_b_ (mm^3^) is volume of the beaker.

### 2.9 Solid density

The solid density ρ_s_ (kg/m^3^) was calculated by filling the 100‐ ml beaker with 50 ml of distilled water and then placing there 3 g sample of rice. The displaced water (volume of the grains) is recorded. The measurement is repeated three times (Shittu, Olaniyi, Oyekanmi, & Okeleye, 2012):

$$\rho_{s}\;=\;\frac{M_{\mathrm{gs}}}{V_{\mathrm{dw}}}$$

where *M*_gs_ (g) is mass of the grain and *V*_dw_ (mm^3^) is volume of displaced water.

### 2.10 Porosity

Porosity *ε* (%) was calculated using results of analysis mentioned above—solid density and bulk density. The formula was described by Jain and Bal (1997):

$$\varepsilon \;=\;100\;\times \;(1-\frac{\rho_{b}}{\rho_{s}})$$

### 2.11 Thousand kernel weight

The 1000 kernel weight m1000 (g) was measured by random selection of one thousand grains of each cultivar and carefully weighed on digital scale Kern 572‐30 with accuracy of 0.001 g following by estimation the final weight in grams. The procedure was repeated five times, and average values were taken (Varnamkhasti et al., 2008).

### 2.12 Color characteristic

Color characteristics of the rice kernels L* (−), a* (−), b* (−) were determined using the spectrophotometer CM‐600d (Konica Minolta Optics, Inc.) based on the CIE laboratory system. The colorimeter was adjusted on D 65—simulation of daylight and the angle of observation was 10°. The measurement was repeated five times with each cultivar, and the data were downloaded from the device to Excel file. Each measurement was recorded in three dimensions: “*L*” (lightness), “*a*” (red/green ratio), and “*b*” (yellow/blue ratio) (Mir et al., 2013).

### 2.13 Optimum cooking time

For the optimum cooking time determination Top (min), it was taken a sample of 5 g from each variety, which was placed in glass Petri dish. The water bath Memmert (Memmert GmbH + Co. KG., Schwabach, Germany) was filled with water till the marked line. Inside of the water bath was placed 250 ml graduated beaker and filled with 100 ml of distilled water. The water bath was brought to the temperature of 100°C, when the water starts to boil. When water reaches the demanded temperature, rice sample was placed into the cylinder and the time started to be recorded. After 10 min of boiling, 3 grains of rice were removed and pressed between two glass plates, in order to examine the gelatinization of the core. Then every other minute, the procedure was repeated, until the rice grains have no more un‐cooked centers. Until at least 9 of 10 grains were properly cooked and then were the rice cooked for another minute to ensure that all the grains are cooked. The optimal cooking time of each cultivar was recorded.

### 2.14 Water uptake ratio

For water uptake ratio determination Wup (−), the water bath Memmert was used. From each rice variety, 18 samples (2 g each) were soaked on water with different temperatures and soaking times. The test temperatures were set on 30, 45, and 60°C and soaking time was set on 30 or 60 min. Each measurement was performed in triplicate. The 2 g sample was placed in 200 ml beaker which was filled with 100 ml of water and inserted into the water bath of certain temperature and soaked for certain time. After the rice was decanted through colander with dense mesh and dried with paper towel, the sample was reweighted and the new weight was recorded. The water uptake ratio was calculated by dividing weight of rice after cooking to initial rice sample weight. It was preceded by recording the weight of the initial raw rice sample and the final rice sample on the electronic scale (Kern 572‐30 with accuracy of 0.001 g) (Shittu et al., 2012).

### 2.15 Rupture force

Rupture force Fr (N) was measured on Texture analyzer MPTest 5.050 (Labortech, Opava, Czech Republic). For each cultivar, it was used 20 grains to perform the measurement. Each grain was placed in the middle of the steel disk along its thickness, and the test was started. After breaking of the grain, the test was stopped and rupture force was recorded.

## 3 RESULTS AND DISCUSSION

The longest cultivar from native varieties was Ozgon Champion with 6.99 mm (the longest from research sample), and shortest was Batken with 6.05 mm. From the imported varieties, the longest rice was Pakistan with 6.53 mm and the shortest Elita Krasnodar Russia with 5.29 mm, which was also the shortest from the whole research sample. Recorded values have been sorted according to FAO classification into long/extra long (2.52–3.10 mm) and bold/slender (1.88–2.13 mm) rice shape (Table 1). Results show clear difference between native and imported varieties (Tables 2 and 3).

**Table 1:** Dimensions and shape classification of the grain

Grain/Rice variety	Length (mm)	Length*	Width (mm)	Shape*
Elita K. Russia	5.29 (0.23)	Medium	2.91 (0.31)	Bold
Ozgon Champion	6.99 (0.34)	Long/extra long	2.94 (0.27)	Bold
Batken	6.05 (0.30)	Long	3.10 (0.17)	Slender (long)
Ozgon Kakyr	6.89 (0.24)	Long	2.91 (0.14)	Bold
Ozgon Uchuk	6.96 (0.47)	Long	2.86 (0.14)	Bold
Pakistan	6.53 (0.45)	Long	2.52 (0.27)	Bold
Kapchygai Kazakhstan	6.00 (0.34)	Long/medium	2.91 (0.19)	Bold
Ozgon Cerza	6.90 (0.48)	Long	2.71 (0.14)	Bold

*Notes*

Values are expressed as mean (standard deviation).

Classification adopted from IRRI (2017).

**Table 2:** Physical characteristic of Kyrgyz rice varieties

Property	Ozgon Champion	Batken	Ozgon Kakyr	Ozgon Uchuk	Ozgon Cerza
Length (mm)	6.99 (0.34)	6.05 (0.30)	6.89 (0.24)	6.96 (0.47)	6.90 (0.48)
Width (mm)	2.94 (0.27)	3.10 (0.17)	2.91 (0.14)	2.86 (0.14)	2.71 (0.14)
Thickness (mm)	1.96 (0.09)	2.13 (0.13)	2.02 (0.15)	2.04 (0.07)	2.01 (0.13)
Equivalent diameter (mm)	3.47 (0.14)	3.43 (0.10)	3.47 (0.07)	3.47 (0.11)	3.37 (0.08)
Surface area (mm²)	31.78 (2.18)	30.34 (1.80)	31.78 (1.34)	31.90 (2.09)	30.38 (1.45)
Sphericity	0.491 (0.02)	0.559 (0.03)	0.498 (0.02)	0.495 (0.02)	0.487 (0.03)
Aspect ratio	0.42 (0.04)	0.51 (0.04)	0.42 (0.03)	0.41 (0.03)	0.40 (0.04)
Volume (mm^3^)	22.02 (2.70)	21.13 (1.91)	21.91 (1.29)	21.84 (2.03)	20.09 (1.32)
Bulk density (g/cm^3^)	0.80 (0.04)	0.82 (0.03)	0.77 (0.02)	0.78 (0.02)	0.77 (0.01)
Solid density (g/cm^3^)	1.26 (0.04)	1.17 (0.05)	1.29 (0.04)	1.18 (0.04)	1.21 (0.05)
Porosity (%)	36.54 (3.47)	30.20 (1.06)	39.83 (1.60)	34.05 (3.25)	36.60 (1.84)
Thousand kernel weight (g)	26.60 (0.32)	24.37 (0.38)	26.57 (0.17)	25.39 (0.16)	27.15 (0.07)

*Notes*

Values are expressed as mean (standard deviation).

Rice varieties are known by their brand name, as no information on their scientific name had been available.

**Table 3:** Physical characteristic of imported rice varieties

Property	Elita Krasnodar Russia	Pakistan	Kapchygai Kazakhstan
Length (mm)	5.29 (0.23)	6.53 (0.45)	6.00 (0.34)
Width (mm)	2.91 (0.29)	2.52 (0.27)	2.91 (0.19)
Thickness (mm)	1.92 (0.08)	1.88 (0.12)	1.95 (0.15)
Equivalent diameter (mm)	3.14 (0.14)	3.16 (0.17)	3.28 (0.14)
Surface area (mm²)	25.35 (1.94)	26.74 (2.82)	27.99 (2.29)
Sphericity	0.592 (0.05)	0.480 (0.02)	0.540 (0.03)
Aspect ratio	0.55 (0.07)	0.39 (0.04)	0.49 (0.03)
Volume (mm^3^)	16.25 (2.14)	16.63 (2.83)	18.61 (2.37)
Bulk density (g/cm^3^)	0.87 (0.01)	0.79 (0.03)	0.84 (0.02)
Solid density (g/cm^3^)	1.41 (0.08)	1.22 (0.03)	1.17 (0.03)
Porosity (%)	38.32 (3.15)	34.84 (2.75)	28.27 (1.84)
Thousand kernel weight (g)	19.67 (0.10)	19.79 (0.28)	21.69 (0.08)

*Notes*

Values are expressed as mean (standard deviation).

Rice varieties are known by their brand name, as no information on their scientific name had been available.

From the analysis of measurement of length, width, and thickness categories can be concluded, that Batken rice variety, local to Kyrgyzstan, has one of the largest physical dimensions from the research sample while imported Pakistan owns one of the smallest dimensions in the research fragment. When measuring the physical properties of rice, grain dimensions are the main characteristics that need to be involved (Bhattacharya, 2011). Most of the authors, while dealing with physical properties of rice establish those basic values (Díaz, Kawamura, & Koseki, 2015; Meena, Vijayalakshmi, & Ravindra, 2010; Oli, Ward, Adhikari, & Torley, 2016; Ravi, Menon, Madhavan, Priyadharshini, & Dhivya, 2014; Smanalieva et al., 2015; Thakur & Gupta, 2006; Varnamkhasti et al., 2008).

Measuring the grain dimensions is first step to characterize the rice shape, and those values are also further used for several dimensional calculations. Smanalieva et al. (2015) measured length, width, and thickness at Ozgon rice variety, in different storage times. We followed same approach, but only the results from longest time of storage were considered, as they were most similar to rice quality in this analysis. The average length of Ozgon rice recorded by Smanalieva et al. (2015) was 6.37 mm (±0.21) which is lower compared to average results of Kyrgyz varieties 6.76 mm (±0.36) in this study. The dimensions for width of grain were 2.80 mm (±0.10) which is significantly similar to our results 2.90 mm (±0.17). The thickness resulted in the study of Smanalieva et al. (2015) 1.87 mm (±0.05) which is slightly lower than result 2.03 mm (±0.11) obtained in this study. Similar results were found by Fofana et al. (2011) or Díaz et al. (2015) who was also measuring wider spectrum of different varieties.

The equivalent diameter varied from 3.37 mm for Ozgon Cerza to 3.47 for Ozgon Champion, Ozgon Kakyr and Ozgon Uchuk in the native cultivars group. In the imported varieties were recorded values from 3.14 mm (Elita K. Russia) to 3.28 mm for Kapchygai Kazakhstan. The result of equivalent diameter corresponds with previous conclusions—hence, the three largest eq. diameters belong to the group of local varieties, and three lowest represent the non‐native cultivar group. Equivalent diameter is next important variable to establish grain characteristic. Mir et al. (2013) with Indian varieties recorded results ranging from 3.60 to 3.79 mm and (Varnamkhasti et al., 2008) measured 3.30 mm and 3.40 mm on cultivars from Iran. Both findings are quite similar to the results of this study.

Surface area values varied from 25.35 mm² (for Elita K. Russia) to 31.90 mm² for Ozgon Uchuk. The cultivars with the lowest surface area are repeatedly the imported varieties—Elita K. Russia, Pakistan, and Kapchygai Kazakhstan, respectively. The cultivars with highest surface area are all the Ozgon varieties represented in the research sample. It is important to mention quite values of standard deviation. Another authors stated results of 34.32 mm² till 43.78 mm² for paddy rice from India (Mir et al., 2013), 39.63 mm² till 49.69 mm² on the new Nigerian rice (Shittu et al., 2012), 13.998 mm² on Indian paddy long‐grain varieties (Thakur & Gupta, 2006), and 25.62 mm² to 38.46 mm² on rice which was studied by (Varnamkhasti et al., 2008) and which was the closest to results of this study. Mohapatra and Bal (2006) mentioned that surface area and thickness of grain are very important for decision about diffusion water during cooking process. Also study published by Juliano (1993) confirms the statement that optimal cooking time for rice correlates with the thickness and surface area of particular grain.

The span of results in sphericity measurement went from 0.48 till 0.59 (0.49–0.56 for local varieties and 0.48–0.59 for imported ones). According to Mohsenin (1986), the sphericity values of rough rice should vary in the range of 0.32–1. In the context of this statement, the results of this analysis are included in the interval and are consistent with Mohsenin's hypothesis. Some other authors as Mir et al. (2013) stated that the paddy Indian rice had its sphericity in range of 0.32–0.53. Díaz et al. (2015) who observed Japanese rough rice (Japonica, Indica and NERICA) documented sphericity to vary from 0.38 to 0.55 and Varnamkhasti et al. (2008) showed the sphericity of Iranian rice in range of 0.37–0.46.

The aspect ratio was found to be highest in Elita Krasnodar Russia (0.55) while lowest in Pakistan (0.39), both belonging to the imported varieties group. The highest aspect ratio of variety of Kyrgyz origin had Batken with 0.51 and the lowest of Kyrgyz group had Ozgon Cerza with 0.40. As was mentioned by Varnamkhasti et al. (2008), the determination of aspect ratio is important due to classification of grain dimensions and the extent of off‐size in market ranking. Their measurement showed values of 0.28–0.29 while for the same variable measured (Mir et al., 2013) for different cultivars of rice aspect ratio of 0.19–0.43. In comparison with those measurements, results of this study range are in upper level.

The volume of the rice grain in fluctuated from 16.25 mm^3^ to 22.02 mm^3^ (local from 20.09 mm^3^ for Ozgon Cerza to 22.02 mm^3^ for Ozgon Champion and imported from 16.25 mm^3^ for Elita K. Russia to 18.61 mm^3^ for Kapchygai Kazakhstan). These results display connection between rice origin and the grain dimensions. The three imported rice varieties represent the lowest values; therefore, we can consider them as the rice varieties with smallest kernels. Varnamkhasti et al. (2008) results of volume of Iranian rice were in range from 13.94 mm^3^ to 26.75 mm^3^. They also mentioned that the volume determination is important for its use for modeling of grain aeration and consequently for all the drying, heating, and cooling devices. Díaz et al. (2015) found values of volume in range from 21.1 mm^3^ to 36.4 mm^3^. Compared to the results of different authors, the volume values from this measurement were significantly lower, particularly due to lower grain dimensions. Quite similar results are provided in the study of Varnamkhasti et al. (2008).

Bulk density describes grain behavior in the dry mass; meanwhile, the solid density is more focused on particular volume the grains take in space, when measured by water test. In this study were recorded values from 0.77 g/cm^3^ to 0.87 g/cm^3^ while the bottom was occupied by two Ozgon cultivars and top was owned by Elita K. Russia as an imported cultivar. This simply explains that the local varieties have longer and less regular shape of the kernel thus they take up larger space. The result of this analysis was comparable to the study of Singh, Kaur, Singh Sodhi, and Singh Sekhon (2005) who measured solid density in range of 0.77–0.87 g/cm^3^ and to results of Meena et al. (2010) with Indian rice range between 0.76 and 0.89 g/cm^3^. Furthermore, Singh et al. (2005) discussed the role of bulk density as an important factor influencing cooking time as well. Varnamkhasti et al. (2008) received the results in range from 0.47 to 0.55 g/cm^3^ adding that bulk density analysis is useful for the design of silos and hoppers for handling and storage of rice as with higher values of bulk density, the rice requires lower packing space. Therefore, in this study, the imported varieties would generally need smaller package for the same amount of rice compared to local varieties (Thakur & Gupta, 2006). The solid density was more balanced within the varieties than bulk density. The solid density values were observed from 1.17 g/cm^3^ (imported and local varieties) till 1.41 g/cm^3^ (imported Elita K. Russia). Shittu et al. (2012) measured in their study 1.06 and 1.28 g/cm^3^. Varnamkhasti et al. (2008) were recording solid density of the rice grains with values between 1.19 and 1.27 g/cm^3^.

Relatively large variability of results of porosity was recorded in this study. Overall porosity varied from 28.27% to 39.83% in the whole sample: 30.20% for Batken—39.83% for Ozgon Kakyr in local varieties and for imported varies K. Kazakhstan (28.27%) and Elita K. Russia (38.32%). As there is no clear pattern, the distribution of porosity among the local and imported varieties is equally intermittent. All the results of other authors have been significantly higher than porosity measured at Kyrgyz‐grown rice varieties. Shittu et al. (2012) measured porosity at two samples with values 45.30% and 57.01%, Thakur and Gupta (2006) recorded 48.06% for their paddy rice, and Varnamkhasti et al. (2008) found results in range of 53.07%–63.33%. Closest value to this study, 45.30%, was measured by Shittu et al. (2012). According to Varnamkhasti et al. (2008), low percentage of porosity, such as the one presented at our test sample, could cause difficulties in active drying process of rice. In case of convective drying with forced draft, the low porosity means that the resistance toward air combustion product is low resulting in slower drying than in case of rice with high porosity.

This analysis displayed the mass weight equivalent of one thousand rice grains, from each cultivar, respectively. The values were in range from 19.67 g for Elita K. Russia to 27.15 g for Ozgon Cerza. As a consequence, the imported varieties could be considered as lighter in weight in comparison with Kyrgyz varieties, as their TKW is visibly lower in all cases (Tables 2 and 3). This pattern validate previous results about smaller grain dimensions and volume; therefore, apparently the weight of grain is lower as well. According to Smanalieva et al. (2015), the Ozgon varieties differed in their TKW from 23.63 to 24.94 g while this value was decreasing with increasing storage time. Those values are in agreement with our study and others authors, such as Mir et al. (2013) who measured TKW in range from 22.23 g to 28.63 g or Díaz et al. (2015) with quite similar results (15.3 – 24.1 g). Also Ravi et al. (2014) measured comparable values in range from 20.6 g to 24.5 g of TKW. According to that article, the thousand kernel weight is also useful parameter to measure the “milling outturn” as a determination of the relative amount of foreign matter in a given volume of paddy rice.

Color characteristic of all rice varieties was examined with spectrophotometer CM‐600d working in the CIE system. Therefore, the values for L* (lightness), a* and b* were examined, respectively. All the color values are noted in Table 4. The whitest of all cultivars was measured to be Kapchygai Kazakhstan with value 72.19 in the L* parameter, while it was followed by Elita Krasnodar Russia with value of L* equal to 69.80. The darkest in light (but not specifically in color) was Ozgon Kakyr with L* corresponding to 37.58. For summary, as the lightest Kyrgyz variety was found Batken and as the darkest Ozgon Kakyr (37.58), while in the group of imported varieties, the lightest was Kapchygai Kazakhstan and the darkest Pakistan with value 56.59. In the context of research sample, the highest a* value reached Ozgon Uchuk (10.17) which puts it on the scale closest to “greenish” color; however, the value in context is still very small. Consequently, the final result sounds that the distribution of color (green or red tones) among all cultivars is equally divided between those two. The lowest a* value, therefore closest to “reddish” color, was Elita K Russia with –0.22. All three imported cultivars have placed in the lowest a* values, thus closest to the “reddish” color from whole sample. According to the parameter b*, the highest value thus yellowness had rice variety Pakistan with 21.12 followed by Ozgon Uchuk with 18.00. On the other hand, the lowest values of parameter b* were measured by Ozgon Cerza (9.65) and Ozgon Champion (10.13). From the previous results can be concluded that most of the varieties (precisely 6 out of 8) have had values of lightness over 50 points; therefore, they are “light” in color. Also two lightest variates come from foreign country; thus, those are probably lighter than local species.

**Table 4:** Color characteristics in CIE laboratory system

Rice cultivars	No. of measurements	L*	a*	b*
Elita K. Russia	5	69.80 (3.14)	–0.22 (0.16)	12.24 (0.34)
Ozgon Champion	5	45.74 (1.80)	9.63 (0.20)	10.13 (0.71)
Batken	5	67.49 (1.52)	3.37 (0.23)	16.95 (0.55)
Ozgon Kakyr	5	37.58 (3.27)	9.98 (1.19)	11.11 (1.87)
Ozgon Uchuk	5	56.28 (3.53)	10.17 (0.82)	18.00 (0.97)
Pakistan	5	56.69 (1.51)	1.61 (0.46)	21.12 (1.04)
Kapchygai Kazakhstan	5	72.19 (1.64)	1.94 (0.25)	12.66 (0.87)
Ozgon Cerza	5	41.79 (1.61)	9.74 (0.46)	9.65 (0.79)

*Note*

The values in brackets are expression of standard deviation.

Oli et al. (2016) were describing three varieties of milled rice grown in Australia. He found color values (L*, a*, and b* values, respectively) 74.66, –0.17, and 16.63 for Reiziq variety and 74.82, –0.33, and 17.31 as the average color characteristic from both low and high head rice yield of Sherpa variety. Also Lamberts et al. (2007) were measuring various de‐husking states of rice and its effect on rice color, while the milled rice had color values approximately (L*, a*, and b*, respectively) 58.7, 5.1, and 24.3. Smanalieva et al. (2015) measured Ozgon with results (L* –66.04, a* –10.76, b* –20.09), which are similar to values of Ozgon Uchuk in this study.

Rupture force of grain is one of the most important quality parameters, which define storage quality, cooking behavior, and also customer preference. Rupture force of grain has been measured, while all data were recorded and later on analyzed. Results of this measurement are relatively divergent. The extreme values in this analysis were fluctuating from 63.47 N in the case of Batken variety (average value from twenty repetitions) to 155.50 N in the case of Ozgon Cerza, which means the difference between bottom value and upper value shows almost 245% increase in rupture force. That is quite unusual considering that large heterogeneity was recorded even within the groups of local and imported varieties. Regarding Kyrgyz varieties, the top value of rupture force was recorded at Ozgon Cerza (155.50 N) and the lowest at Batken (63.47 N). In the imported rice cultivars reached the highest rupture force Pakistan with 130.28 N and the lowest rupture force got Elita Krasnodar Russia with 66.85 N. The overall result of the rupture force measurement showed that there is no direct linkage between rupture force and origin of eight milled rice cultivars present in the research sample. The variation in the rupture force could be caused by different compact arrangement of the starch granules in the center of the kernel (Mir et al., 2013). In terms of the classification, we can claim that the Ozgon cultivars, popular by local people, have the top rupture force from the examined varieties. Similar results recorded Fofana et al. (2011) with rice rupture force range from 59 N to 122 N and Mir et al. (2013) with range from 72.99 N to 133.48 N.

**Figure 1:**
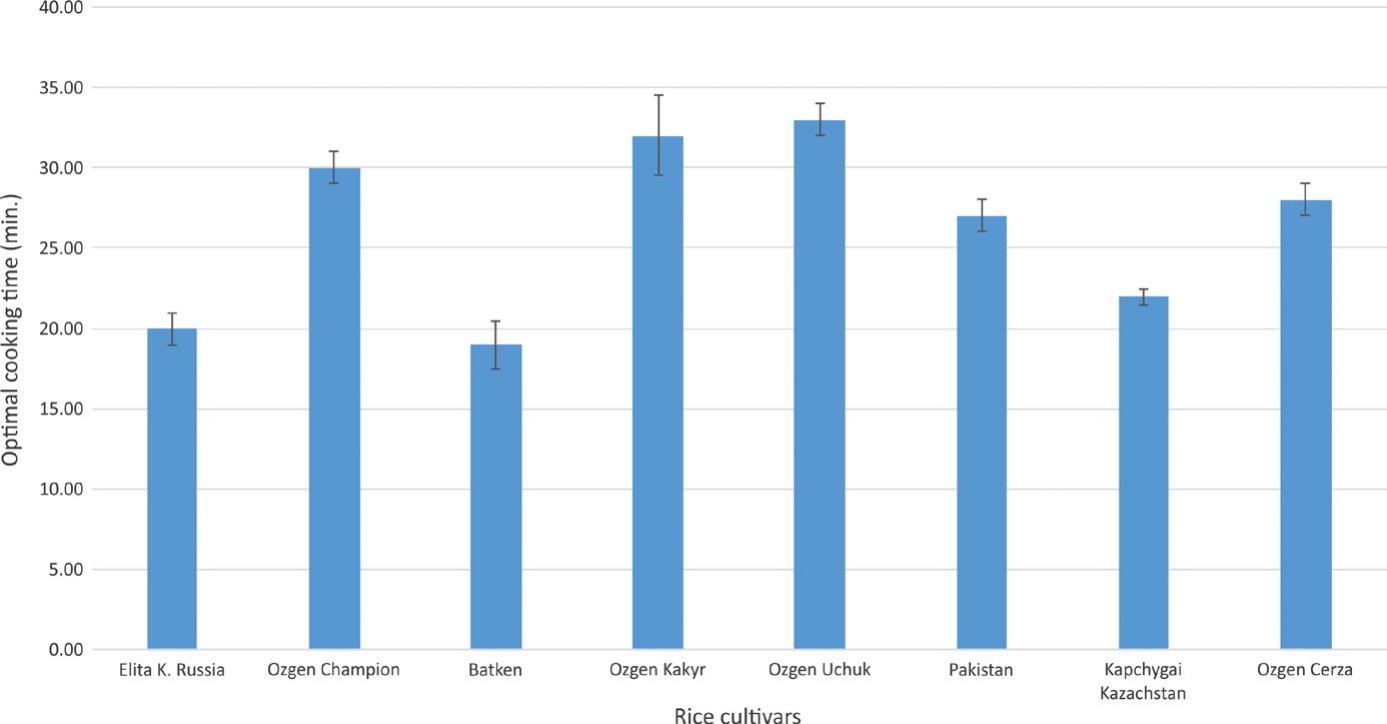
Optimal cooking time of selected rice varieties

Determination of cooking time of the rice varieties is one of two cooking characteristics examined during this study. Cooking time is very important information in relation to consumer preferences and also certain types of rice cultivars and their utilization. Figure 1 shows bellow summary of optimal cooking times of all cultivars. The outer borders of this measurement results belong to Ozgon Uchuk (with 33 min) and to Batken (with 19.33 min) which makes the difference between the extremes over 13 min. In the Kyrgyz, traditional varieties' group keeps the average cooking time around 30 min while the imported varieties are generally shorter in optimal cooking time. According to Yadav and Jindal (2007), the cooking process is an important operation and the length of cooking and final texture of the rice kernels is crucial for customers to select the optimal variety for their consumption. As stated by Singh et al. (2005), main factors affecting variations in cooking quality of rice are genetic and environmental. As the differences between imported and local Kyrgyz varieties are significant, the results will be compared respectively to this frame. Similar results to optimal cooking time of Kyrgyz varieties (with exception of Batken) measured by Deepa, Singh, and Naidu (2008) who got results in range of 30 to 38 min. Also Jinorose, Prachayawarakorn, and Soponronnarit (2014) measured average cooking time of rice around 30 min, which agree with results of this study. Quite similar results as imported varieties grown in Kyrgyzstan were found by Fofana et al. (2011) with range of cooking time from 17 to 26 min compared to 20–27 min of imported varieties of this study.

**Figure 2:**
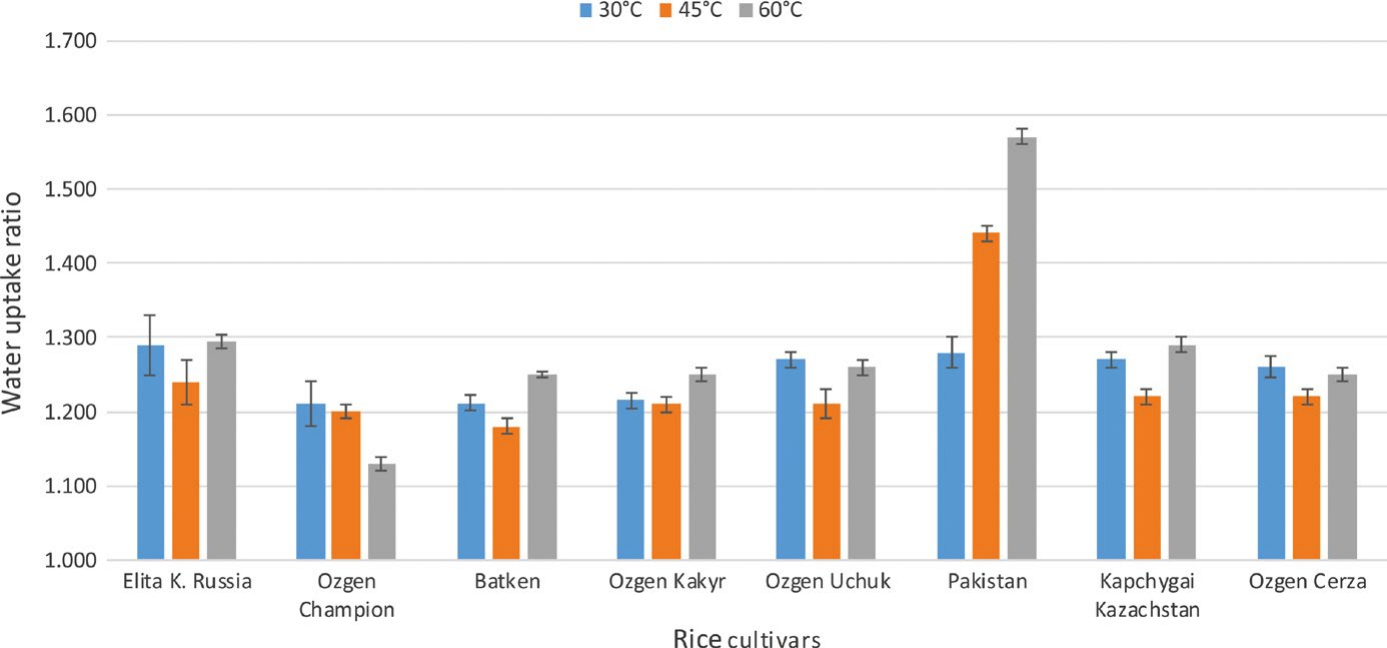
Water uptake ratio of selected rice varieties

Water uptake measurement was involved in the set of analysis, as it pictures other textural characteristic according to rice rather than simply cooking. The water uptake ratio was registered for three temperatures: 30, 45, and 60°C, all in two time segments: 30 min and 60 min. The results of 30‐min soaking are shown in Figure 2. In the first part, where rice was soaked for 30 min at different temperatures could be observed significant difference between behavior of Pakistan rice and all the other varieties. In the conditions of rising temperatures and stable time of soaking, the Pakistan and Ozgon Kakyr were the only cultivars which reacted with steadily increasing amount of water uptake (Figure 2). On the contrary, the rest of cultivars behaved considerably different, when they all (with exception of Ozgon Kakyr) decreased in water uptake ratio at 45°C and again rise (with exception of Ozgon Champion) at 60°C but only to the level of 30°C values or slightly above that. Pakistan cultivar greatly stands out from the test sample with its absorption characteristics.

In the measurement with longer time period of soaking, 60 min, the results were actually similar, with slightly larger water uptake ratio values. Water uptake ratio was measured by several authors (Fofana et al., 2011; Meena et al., 2010; Mizuma, Kiyokawa, & Wakai, 2008; Mohapatra & Bal, 2006; Singh et al., 2005; Thakur & Gupta, 2006; Yadav & Jindal, 2007). However, many of them performed the measurement with different methodologies compared to we used in this study. Only Shittu et al. (2012) used similar temperatures (30, 45, and 60°C) for water uptake ratio measurement. The collective also measured unusual behavior of rice at 45°C which they attribute to some microstructure details that are yet to be studied for the different rice varieties. Nevertheless, the results of their study showed gradually increasing values with increasing temperature unlike this study. Yet Shittu et al. (2012) recorded some nontypical behavior of rice as well. Unusual reduction in hydration rate was observed with paddy rice of ITA 150, ITA 301, and WAB 450 as soaking temperature was increased from 45 to 60°C. The reduction in hydration rate may be partly due to slight swelling of the grains which could have led to decrease in porosity of the material and consequently reduction in water absorption rate. The water uptake in this study did not showed large increase between 30 and 60 min; therefore, we can assume that the main factor influencing water uptake is temperature rather than time. Also the temperature levels used in this study (30, 45, and 60°C) were probably sufficient to model the precooking rice soaking. However, with an emphasis to the results of this measurement, those temperatures are too low to show significant increase in water uptake ratio. According to Mohapatra and Bal (2006), rice with higher water binding capacity normally yields soft textured cooked product, opposite to the lower binding where the grains remains more firms and structured. As the result we can claim, that the Kyrgyz rice is after cooking staying more firm and therefore probably more suitable for the local traditional dish “plov” compared to imported variety of Pakistan, which end up in soft textured product with different use in the national cuisine.

The rice grain characteristics obtained by this study are important for the producers and consumers in Kyrgyzstan and will provide guidance in terms of breeding, processing, and retail of those rice varieties. In the future studies, it might be important to compare the Kyrgyz varieties with quite wider sample of already known and well‐described rice varieties around the world. In particular, the comparative tests with rice varieties with possible similar use such as varieties used for risotto dish (carnaroli, arborio) or paella dish (senia, bomba) might be very interesting. Such findings may extend the pool of knowledge of less investigated rice varieties from Kyrgyzstan and thus may be interesting for their international marketing.

## 4 CONCLUSION

This study represents extensive collection of information on physical properties of rice cultivars grown and consumed in Kyrgyzstan, Central Asia. The research had shown that the physical properties, texture characteristics, and cooking characteristics among all cultivars differ significantly. The broad variation applied to grain length and shape (from medium to long and from bold to slender) which is particularly important information for designing milling machinery and all kinds of optimization in the postharvest operations (handling, processing, and packaging) while avoiding any loses and damage. According to grain dimensions and physical characteristics (equivalent diameter, surface area, and volume) had been proven that varieties with non‐native origin differ greatly from the traditional Kyrgyz varieties, and in all those parameters, they showed significantly lower values. The sphericity, aspect ratio, solid density, and porosity showed results with wide range of values; however, in comparison between local and imported group of cultivars could not been declared any assumptions as the values showed nonconsistent patterns. The imported varieties had significantly higher values in measurement of bulk density which confirmed the lower values of grain dimensions. At the same time, the thousand kernel weight resulted in clear pattern where local cultivars reached upper level in weight of their kernels. Color of the grain was estimated based on CIE laboratory system and the rupture force established showed wide range of recorded values (63.47–155.50 N); however, neither one of the cultivar sections was dominant in the test. That information is useful for discovering the suitable use for each cultivar. Optimal cooking time showed that imported varieties needed lower interval for full grain cooking compared to the local Kyrgyz varieties. Water uptake ratio came up with results strictly separating the soaking characteristics of Pakistan rice from the rest of varieties. The rice grain characteristics obtained by this study are important for the producers and consumers in Kyrgyzstan and will provide guidance in terms of breeding, processing, and retail of those rice varieties.

## AUTHOR CONTRIBUTIONS

Martina Nadvornikova provided most of the analysis and drafted the manuscript; Jan Banout designed the study, coordinated all analysis, finalized, and drafted the manuscript; David Herak suggested appropriate methods and contributed to results interpretation; Vladimir Verner collected the material, assisted in study design, and contributed to finalization of the manuscript. All authors read and approved the final manuscript.
